# Glycogen in the Treatment of Diabetic Albuminuria

**Published:** 1904-08

**Authors:** 


### ABSTRACTS.

---

### Glycogen in the Treatment of Diabetic Albuminuria.

M. LAUMONIER, (*Bulletin General de Therapeutique,* January 15, 1904,) calls attention to the value of glycogen in the treatment of diabetic albuminuria. He recalls the fact that last year he was led to conclude that the hepatic cell in diabetics seemed to have lost the power of fixing glycogen in its cytoplasm. His researches have since been confirmed by Monier, of Liege. This brings to mind the fact that Frerichs many years ago, having obtained by trocar from a diabetic patient a parcel of hepatic tissue, ascertained microscopically the absence of glycogen in the hepatic cell. Albuminuria is a grave complication of diabetes and requires active treatment. Unfortunately, this symptom and diabetes are, as it were, antagonistic, the former requiring a milk diet, or at least a diet rich in hydrocarbons; the second, on the contrary, demanding a diet from which are excluded as much as possible starches and sugars. Again, considering the condition of the kidneys in diabetics, certain remedies now used, antipyrin and other toxics, cannot be employed. The writer found that the methodic use of glycogen seemed to avoid this difficulty, and mentions a number of cases with charts to sustain his point. The dose administered began, as a rule, with one gramme (15 grains) in the course of the day.—*Monthly Cyclopedia,* April, 1904.

